# 255 Evaluation and Comparison of Government-Funded School-Based Youth Physical Activity Counseling Initiatives in Finland for the 2024-2025 Academic Year

**DOI:** 10.1093/eurpub/ckae114.279

**Published:** 2024-09-26

**Authors:** Iiris Kolunsarka

**Affiliations:** University of Jyväskylä, Finland

**Keywords:** physical activity counseling, youth, governmental initiatives

## Abstract

**Introduction:**

Finland’s newly elected government launched their four-year programme titled “A Strong and Committed Finland.” Among its objectives is a significant emphasis on increasing physical activity across all age groups. To this end, the government introduced the 'Finland on the Move' initiative, dedicated to promoting an active lifestyle and improving the physical well-being of Finns. The initiative is supported by an annual budget of twenty million euros, and it consists of sixteen actions, with six specifically aimed at promoting physical activity among youth. One key action focuses on enhancing physical activity in schools, particularly through school-based youth physical activity counseling.

**Project aim:**

This research project aims to evaluate and compare the government-funded school-based youth physical activity counseling initiatives implemented in Finland during the 2024-2025 academic year. The project seeks to answer the following research questions:

a) How is physical activity counseling conducted in Finnish schools?

b) How do participants benefit from the counseling?

**Methods:**

The evaluation will begin with an analysis of publicly available program abstracts. Additionally, interviews and questionnaires will be conducted during and after the 2024-2025 academic year to gather comprehensive data.

**Results (Primary):**

A total of 123 cities received funding for promoting physical activity in schools, with 74 of these cities incorporating physical activity counseling into their project plans. The protocols for counseling varied significantly. Some cities will hire fitness instructors or counselors to work directly in schools, while others aim to create pathways for youth to access services provided by city-employed physical activity counselors. Certain projects focus on family-oriented physical activity counseling or lifestyle counseling, although nearly all target youth with low fitness and physical activity levels.

**Conclusion:**

By identifying effective physical activity counseling strategies, this research project can help schools and cities implement best practices that encourage more active lifestyles among youth. Moreover, the findings from this research project can provide valuable insights for policymakers. Understanding which initiatives are most effective allows for better allocation of resources and more informed decisions about future funding and program development.

